# Difficulty accessing condoms because of the COVID-19 pandemic reported by gay, bisexual and other men who have sex with men in the UK: findings from a large, cross-sectional, online survey

**DOI:** 10.1177/09564624231160804

**Published:** 2023-03-21

**Authors:** Jack RG Brown, David Reid, Alison R Howarth, Hamish Mohammed, John Saunders, Caisey V Pulford, Dana Ogaz, Gwenda Hughes, Catherine H Mercer

**Affiliations:** 1 Institute for Global Health, 4919 University College London, London, UK; 2 The National Institute for Health Research Health Protection Research Unit in Blood Borne and Sexually Transmitted Infections at University College London in Partnership with the UK Health Security Agency, London, UK; 3 Blood Safety, Hepatitis, STIs and HIV Division, 371011 the UK Health Security Agency, Colindale, UK; 4 Department of Infectious Disease Epidemiology, London School of Hygiene and Tropical Medicine, London, UK

**Keywords:** sexual health, contraception, condoms, MSM, COVID-19, gender-diverse

## Abstract

**Background:**

COVID-19 restrictions severely reduced face-to-face sexual health services, an important access point for condoms. We examine whether gay, bisexual and other men who have sex with men (GBMSM) in the UK had difficulty accessing condoms during the first year of the pandemic, and if so, which groups were most affected.

**Methods:**

Questions about difficulty accessing condoms were asked as part of a short, online cross-sectional survey of GBMSM undertaken November/December 2021, recruited via social media and Grindr. Eligible participants were UK-resident GBMSM (cis/trans/gender-diverse person assigned male at birth [AMAB]), aged ≥16 years who were sexually active (reported sex with men in the last year). Multivariable logistic regression was used to examine if and how reporting this outcome varied by key sociodemographic, health and behavioural factors independent of the potential confounding effect of numbers of new male sex partners.

**Results:**

Of all participants (*N* = 1039), 7.4% (*n* = 77) reported difficulty accessing condoms due to the pandemic. This was higher among younger GBMSM (aged 16–29 years vs. ≥45; 12.8% vs. 4.9%; aOR: 2.78); trans/gender-diverse AMAB participants (vs. cis gender males; 24.4% vs. 6.6%; aOR = 4.86); bisexually-identifying participants (vs. gay-identifying; 11.1% vs. 6.5%; aOR = 1.78); and those without degree level education (vs. having a degree; 9.8% vs. 5.6%; aOR = 2.01).

**Conclusions:**

A minority of sexually active GBMSM reported difficulty accessing condoms because of the pandemic, however, this was more common amongst those who already experience a disproportionate burden of poor sexual health. Interventions are needed to address these inequalities in accessing this important primary STI/HIV prevention measure.

## Key messages

1. Condoms remain effective for primary STI/HIV prevention so recommendations state that condoms should be distributed at venues accessed by those at higher STI/HIV risk, including some GBMSM.
2. Using a large, community-based online survey we explored how the COVID-19 pandemic impacted on GBMSM’s ability to access condoms when needed and examined associated factors.
3. A minority of sexually-active GBMSM reported difficulty accessing condoms because of the pandemic, but those groups who already experience a disproportionate burden of poor sexual health were more likely to do so.

## Introduction

HIV diagnoses in the UK amongst gay, bisexual and other men who have sex with men (GBMSM) have been declining since 2014 due to the scale up of HIV combination prevention (testing, pre-exposure prophylaxis [PrEP] and treatment as prevention)^1^. In contrast, bacterial STI diagnoses among GBMSM have been rising, and may, in part, be attributed to behaviour changes including increasing numbers of condomless anal sex (CAS) partners.^2^ Condoms remain an effective component of primary STI and HIV preventive strategies. Recommendations by the National Institute for Health and Care Excellence (NICE) state the importance of targeted condom distribution in places visited by those who are at higher STI/HIV risk or infrequent users of condoms eg, sex on premises venues, bars, nightclubs, sexual health services (SHS), and youth groups.^3^

There is a national network of open access specialist sexual health services across the UK. These are free, confidential, and open to anyone without the need to be resident in the local area, registered with or referred by a primary healthcare physician. On 23 March 2020, the UK announced its first national lockdown in response to the growing SARS-CoV-2 (COVID-19) pandemic.^4^ Consequently, SHS rapidly reconfigured: in-person asymptomatic screening and walk-in appointments were suspended and patients directed online. Many other ‘in-person’ services and social venues (including those for targeted condom distribution to GBMSM) were also closed. COVID-19-related social restrictions in the UK fluctuated over the next 15-months until the removal of most social restrictions on 19 July 2021.^5^ Levels of reported sexual behaviour also fluctuated in line with social restrictions, although a significant minority of GBMSM continued to report sexual risk behaviour during periods of tighter social restrictions eg, over one-quarter (27.6%) of GBMSM in a community-based survey reported multiple CAS partners during the UK’s third national lockdown.^6^ Given the differing levels of access to in-person health services and social venues between March 2020 and July 2021, we explore how the pandemic impacted on GBMSM’s ability to access condoms when needed and examined associated factors.

## Methods

### Study design

The ‘Reducing inequalities in Sexual Health’ (RiiSH)-COVID surveys are repeat, cross-sectional online community surveys, fielded during different stages of the pandemic. This report used the fourth iteration of the survey, fielded 22 November–12 December 2021, after most social restrictions had ended. The fourth survey included questions on participants’ need for, and access to, condoms, at any time, during the pandemic (beginning at the first national lockdown 20 March 2020).

### Setting and sampling

Participants were recruited from social networking sites (Facebook, Twitter, Instagram) and the geospatial dating application Grindr. Adverts on these sites and applications directed individuals to the anonymous online survey. The first questions assessed eligibility, defined as: UK resident; aged ≥16 years; men (cis/trans gender), transwomen, or gender-diverse people assigned male at birth (AMAB); reporting sex in the past year with a man (cis/trans gender) or gender-diverse person AMAB. The survey took on average 10 minutes to complete. Online consent was obtained from all participants. No financial incentive was offered.

### Data collection

The RiiSH-COVID survey was administered using SNAPSurvey software and included questions on sexual behaviour, SHS use, HIV PrEP use, and access to condoms, specifically:

“*Have you ever needed to use a condom but didn’t because you couldn’t get hold of any?*”

Participants who responded “*Yes*” were then asked:

“*Was there a time since the start of the first lockdown (23 March 2020) when you needed to use condoms but didn’t because you couldn’t get hold of any because of the pandemic*?”

The primary outcome was those who responded “*Yes*”

Behavioural questions referred to a lookback period, from August 2021 when most remaining COVID-19 restrictions were lifted (sometimes referred to as ‘freedom day’) until November/December 2021.

### Data analysis

We used Stata (V.17) to calculate the percentage reporting the primary outcome. We then examined how this varied by key sociodemographic, health-related and behavioural variables. Given the strong correlation with the number of new male sex partners reported, reflecting greater opportunities and need to use condoms, we used multivariable logistic regression to adjust for this variable and to identify variables independently associated with the outcome. Statistical significance was set at *p* < 0.05.

### Ethical approval

UCL Research Ethics Committee approved RiiSH-COVID survey rounds (ref:9155/001).

### Patient and Public involvement

Patients and members of the public were involved at several stages of the original RiiSH study upon which the RiiSH-COVID survey was based,^7^ including the design and delivery of the study. We received input from GBMSM who attended sexual health clinics across England on our draft fieldwork documentation, in terms of its comprehension, feasibility to complete (e.g., around recall burden), and acceptability of question topics and wording. We subsequently worked with GBMSM attending sexual health clinics to get their feedback on our online questionnaire when we adapted it for implementation during the COVID-19 pandemic (RiiSH-COVID), the images used to promote the online survey across different digital channels, and the specific social media and dating apps that we proposed to use. We worked with a sexual health charity who promoted the online surveys and who subsequently advised us on some bespoke analyses that they used to inform their outreach activities. We continue to seek patient and public involvement as we disseminate our findings and develop new projects that build on RiiSH-COVID.

## Results

### Participants’ characteristics

Over half of eligible participants (*N* = 1039) were recruited through social media (55.6%), with the remainder recruited from Grindr (Appendix 1). Participants had a median age of 41 years (IQR: 31–51; Range: 17–81). The majority identified as cisgender male (95.7%), white (88.1%), gay (80.9%), resident in England (85.6%), with around three-quarters (76.3%) born in the UK. More than half (56.8%) reported having a degree, and a majority (75.7%) reported having some form of employment. Over one-third of participants (39.4%) lived alone and one-third (33.3%) lived with their partner(s). Around one in 10 participants (11.6%) reported living with HIV.

### Reporting difficulty accessing condoms because of the pandemic

Altogether, 7.4% reported difficulty accessing condoms since the start of the pandemic (55.8% of GBMSM who reported ever having difficulty accessing condoms), attributing this to the ongoing pandemic.

### Factors associated with reporting difficulty accessing condoms because of the pandemic

In multivariable analyses, the following remained significantly associated with the outcome: Younger GBMSM (aged 16–29 years vs 45 and over) were more likely to report pandemic-related access problems (aOR: 2.78) (Table 1), as were participants who reported a gender identity other than cisgender male (vs. cisgender male) (aOR: 5.66), bisexually-identifying participants (vs. gay-identifying; aOR: 1.91), and those without degree level education (vs degree or higher; aOR: 1.91). GBMSM reporting PrEP use in the lookback period (vs. no PrEP use) were less likely to report difficulties accessing condoms (aOR: 0.54).

**Table 1.** Sociodemographic, health and behavioural factors (reported in the lookback) associated with reporting difficulty accessing condoms since the start of the COVID-19 pandemic in the UK (23 March 2020), because of the ongoing pandemic.

	Total % (*n*)	% reporting difficulty (*n*)	uOR (95% CI)	*p*-value	aOR (95% CI)†	*p*-value
Reporting difficulty accessing condoms because of the pandemic						
All	100.0 (1039)	7.4 (77)	-	-	-	-
Sociodemographics						
Age (years)						
45+	41.0 (426)	4.9 (21)	1	0.002	1	0.002
30–44	37.2 (386)	7.0 (27)	1.45 (0.81–2.61)		1.45 (0.80–2.63)	
16–29	21.9 (227)	12.8 (29)	2.82 (1.57–5.08)		2.78 (1.54–5.02)	
Gender identity	—	—	—	—	—	—
Cisgender male	95.7 (994)	6.6 (66)	1	<0.001	1	<0.001
All other gender groups*	4.3 (45)	24.4 (11)	4.55 (2.20–9.39)		5.66 (2.67–12.02)	
Sexual identity	—	—	—	—	—	—
Gay	80.9 (841)	6.5 (55)	1	0.029	1	0.017
Bisexual**	19.1 (198)	11.1 (22)	1.79 (1.06–3.01)		1.91 (1.12–3.26)	
Ethnicity	—	—	—	—	—	—
White	88.1 (915)	7.0 (64)	1	0.167	1	0.159
All other ethnic groups***	11.9 (124)	10.5 (13)	1.56 (0.83–2.92)		1.58 (0.84–2.97)	
Born in the UK?	—	—	—	—	—	—
Yes	76.3 (793)	6.8 (54)	1	0.186	1	0.296
No	23.7 (246)	9.4 (23)	1.41 (0.85–2.35)		1.32 (0.79–2.20)	
Highest educational qualification	—	—	—	—	—	—
Degree or higher	56.8 (590)	5.6 (33)	1	0.011	1	0.007
Below degree	43.2 (449)	9.8 (44)	1.83 (1.15–2.93)		1.91 (1.19–3.07)	
Health-related factors						
HIV-status	—	—	—	—	—	—
Negative/unknown	88.5 (919)	7.2 (66)	1	0.436	1	0.462
Positive	11.6 (120)	9.2 (11)	1.30 (0.67–2.55)		1.29 (0.66–2.52)	
PrEP use in the lookback period (if HIV-neg/unknown)	—	—	—	—	—	—
No	68.1 (626)	7.8 (49)	1	0.269	1	0.048
Yes	31.9 (293)	5.8 (17)	0.73 (0.41–1.28)		0.54 (0.29–0.99)	
Life satisfaction level	—	—	—	—	—	—
Medium–very high	82.7 (856)	6.7 (57)	1	0.067	1	0.057
Low	17.3 (179)	10.6 (19)	1.66 (0.96–2.87)		1.71 (0.98–2.96)	
Self-reported STI symptoms (in the lookback)	—	—	—	—	—	—
No symptoms	81.2 (844)	6.5 (55)	1	0.024	1	0.057
One or more symptoms	18.8 (195)	11.3 (22)	1.82 (1.08–3.07)		1.68 (0.99–2.87)	
Sexual behaviour in the lookback period						
No sex	8.2 (85)	5.9 (5)	1	0.399	1	0.424
Physical sex (no anal)	13.5 (140)	5.0 (7)	0.84 (0.26–2.74)		0.41 (0.11–1.61)	
Anal sex	78.3 (814)	8.0 (65)	1.39 (0.54–3.55)		0.60 (0.18–1.96)	

^†^ Adjusted for reported number of new male sex partners (in the lookback period).

^*^Including: Trans male (*n* = 16); Trans female (*n* = 3); Other gender identity (*n* = 26).

^**^Including: Bisexual (*n* = 145); Straight (*n* = 6); Other (*n* = 47).

^***^Including: Black (*n* = 16); Asian (*n* = 58); Mixed or other ethnic identity (*n* = 50).

## Discussion

This large, community-based survey of GBMSM in the UK found that a minority (7.4%) of participants reported difficulty accessing condoms when needed, because of the pandemic. After adjusting for the number of new male sex partners (which attempts to take account of differing ‘need’ for, and opportunity to use, condoms), the reporting of difficulties accessing condoms varied, and was most common among groups who already experience poorer sexual health, including younger GBMSM, gender and sexual minorities and those with less education. However, those currently accessing PrEP were less likely to report access problems perhaps reflecting better access opportunities.

## Comparison with other literature

The Natsal-COVID study used a quasi-representative sample of the UK population and estimated that 36.8% (95% confidence interval (CI): 27.8%–46.8%) of GBMSM had had difficulty accessing condoms because of the pandemic, five times higher than our figure of 7.3% among GBMSM.^8^ However, the Natsal-COVID sample included a smaller number of GBMSM (*n* = 183), reflecting how it was a study of the general population, and defined GBMSM differently (any same-sex experience in the past 5 years). Indeed, it is worth noting that Natsal-COVID’s estimate was considerably lower at 4.9% (95% CI: 2.1%–11.1%) among the 117 participants who identified as gay, which is more in line with our study’s estimate. As we observed, gay-identifying MSM were considerably less likely to report access difficulties than bisexual-identifying MSM, perhaps reflecting greater awareness of how to access condoms, their use and protective possibilities among gay-identifying MSM. It is interesting to note that this general population study found, as we did, that younger GBMSM (aged 18–29 years) were more likely to report difficulty accessing condoms because of the pandemic (50.4%).^8^ This age effect suggests a continuation of inequalities that preceded the COVID-19 pandemic,^9^ perhaps reflecting how knowledge of, and resources to access condoms, increases with age. However, we are unsure as to the cumulative effect of the pandemic on the magnitude of associations with age and difficulties accessing condoms.

## Strengths and limitations

This large community-based survey of GBMSM in the UK complements national surveillance data on SHS attendees. This enables us to make comparisons on risk behaviours and testing need in GBMSM who do and do not access SHS. The data also complement and contextualise the findings from other studies with smaller sample sizes, like Natsal-COVID.^8^

However, there are limitations. As a cross-sectional survey, associations between variables can be bidirectional and therefore we cannot infer causality. The timeframe for our primary outcome refers to a period when social restrictions were in place across the UK, but the lookback for our behavioural variables was approximately 4 months when social restrictions had ended in the UK, which we use as a proxy measure for behaviour during the COVID-19 pandemic. We acknowledge recall bias in the study and that reported behaviour may not be a true reflection. We tried to word questions that reduced participant recall burden. Online non-random recruitment through social media and dating applications will exclude GBMSM who do not use these platforms, are not seeking new sexual partners, and/or do not have Internet access, limiting the generalisability of our findings to all GBMSM. Finally, given the wording of the question, we cannot make inferences as to the extent to which SHS reconfiguration due to COVID-19, including reduced in-person access to SHS,^6^ played a role in some GBMSM having difficulties accessing condoms during this time. It is also important to recognise the subjectivity surrounding the concepts of ‘needing to use condoms’ and also ‘because of the pandemic’ as per our question.

Given the small number of migrants and participants from ethnic minority groups, we needed to categorise country of birth and ethnicity as binary variables thereby overlooking substantive differences in sexual health within these groups.^10^ Likewise, as the majority of participants were cis gender GBMSM we were unable to make meaningful inferences on barriers to access, and the sexual health needs of, gender minorities.

## Implications

Rather than exacerbating or altering the dynamics of inequalities in condom access, access inequalities seem to be unaffected by social restrictions. However, we cannot comment on any cumulative affects the pandemic has had on the magnitude of difficulty these groups have faced, as we lack any comparative pre-COVID data. Clearly, further efforts are needed to reduce barriers to accessing condoms. As SHS face further reconfiguration post-pandemic, it is opportune to explore convenient and effective approaches for improving condom access, which continues to be a mainstay of STI prevention, such as adaptation of remote condom provision and/or integration with remote testing services.

## Appendix 1 Participants’ characteristics, by recruitment site

**Appendix 1 Participants’ characteristics, by recruitment site.**

	2021 all participants (*N* = 1039) % (*n*)	2021 participants recruited through social media (*N* = 567) % (*n*)	2021 participants recruited through grindr (*N* = 452) % (*n*)	*p*-value *for differences in samples by recruitment site*
Participants’ characteristics				
Median age (years) (IQR; range)	41 (31–51; 17–81)	42 (31–52; 17–81)	39 (31–50; 17–77)	—
Age category (years)	—	—	—	—
16–29	21.9 (227)	21.7 (123)	22.8 (103)	0.024
30–44	37.2 (386)	33.9 (192)	40.9 (185)	
45 and over	40.9 (425)	44.4 (251)	36.3 (164)	
Gender identity	—	—	—	—
Cis male	95.7 (994)	96.1 (545)	95.1 (430)	0.671
Trans male	1.5 (16)	1.6 (9)	1.6 (7)	
Trans female	0.3 (3)	0.4 (2)	0.2 (1)	
All other gender identities	2.5 (26)	1.9 (11)	3.1 (14)	
Ethnicity	—	—	—	—
White (inc. white minorities)	88.1 (915)	90.5 (513)	84.7 (383)	0.005
Black	1.5 (16)	0.5 (3)	2.9 (13)	
Asian	5.6 (58)	4.6 (26)	7.1 (32)	
Mixed and/or other ethnic groups	4.8 (50)	4.4 (25)	5.3 (24)	
Sexual identity	—	—	—	—
Gay	80.9 (841)	86.2 (489)	74.6 (337)	<0.001
Bisexual	14.0 (145)	9.9 (56)	19.3 (87)	
Straight	0.6 (6)	0.7 (4)	0.4 (2)	
All other sexual identities	4.5 (47)	3.2 (18)	5.8 (26)	
Country of residence in the UK	—	—	—	—
England	85.6 (889)	85.7 (486)	85.2 (385)	0.098
Scotland	7.5 (78)	6.9 (39)	8.6 (39)	
Wales	4.7 (49)	5.8 (33)	3.3 (15)	
Northern Ireland	2.2 (23)	1.6 (9)	2.9 (13)	
Born in the UK	—	—	—	—
Yes	76.3 (793)	77.1 (437)	75.4 (341)	0.543
Highest educational qualification	—	—	—	—
Degree or higher	56.8 (590)	59.1 (335)	53.5 (242)	0.076
Currently employed	—	—	—	—
Yes	75.7 (786)	76.7 (435)	75.0 (339)	0.523
Living alone	—	—	—	—
Yes	39.4 (409)	35.8 (203)	43.1 (195)	0.017
Living with partner(s)	—	—	—	—
Yes	33.3 (346)	38.3 (217)	26.6 (120)	<0.001
Anxiety level	—	—	—	—
High	36.1 (372)	35.7 (202)	36.3 (162)	0.835
Life satisfaction level	—	—	—	—
Low	17.3 (179)	15.7 (89)	19.4 (87)	0.120
Living with a HIV diagnosis	—	—	—	—
Yes	11.6 (120)	12.2 (69)	10.6 (48)	0.441
PrEP use (in the lookback) if HIV-negative/unknown	—	—	—	—
Yes	31.9 (293)	29.3 (146)	34.4 (139)	0.102
